# Burden, spectrum and associated factors of ocular disease among incarcerated populations in sub-Saharan Africa: a systematic review and meta-analysis

**DOI:** 10.3389/fmed.2026.1969779

**Published:** 2026-10-23

**Authors:** Josephine Ampong, Isaiah Osei Duah Junior, Bismark Beesi Coffie, Kelvin Oppong Kyeremateng, Emmanuel Tettey Doku, Eric Tettey Ashong, Kwesi Sewe, Loretta Addo-Danso, Albert Kwadjo Amoah Andoh, David Ben Kumah, Hornametor Yao Afake, Werner Eisenbarth, Kwadwo Owusu Akuffo

**Affiliations:** 1 Department of Optometry and Visual Science, College of Science, Kwame Nkrumah University of Science and Technology, Kumasi, Ghana; 2 Department of Psychology, John R. and Kathy R. Hairston College of Health and Human Sciences, North Carolina Agricultural and Technical State University, Greensboro, NC, United States; 3 University of Ghana Library System, University of Ghana, Accra, Ghana; 4 Department of Sociology and Criminal Justice, University of Delaware, Newark, DE, United States; 5 National Eye Care Unit, Ghana Health Service, Accra, Ghana; 6 Department of Applied Science and Mechatronics, HM Hochschule München University of Applied Sciences, Munich, Germany

**Keywords:** blindness, eye diseases, incarceration, ocular morbidity, ophthalmology, prison inmates, resource-limited settings, visual impairment

## Abstract

**Background:**

Ocular morbidity remains poorly characterized among prisoners; this systematic review and meta-analysis quantified its prevalence, spectrum and associated factors across sub-Saharan Africa (SSA).

**Methods:**

This review followed a published Preferred Reporting Items for Systematic Review and Meta-Analysis (PRISMA) Protocols (PRISMA-P) and searched eight databases using prespecified terms, with study selection guided by PRISMA. Data were standardized and synthesized using random-effects meta-analysis to estimate pooled prevalence with 95% confidence intervals (CIs), with heterogeneity assessed using *I*^2^ and sensitivity and condition-specific analyses used to assess variation.

**Results:**

Six studies comprising 3,361 participants from four SSA countries, Nigeria, Uganda, Tanzania and Kenya, met the inclusion criteria. The pooled prevalence of ocular morbidity was 43.1% (95% CI: 12.4–77.1%; *I*^2^ = 99.2%). Conjunctivitis was the most prevalent condition (23.9%; 95% CI, 10.7–40.1%), followed by refractive error (19.5%; 95% CI, 12.5–27.5%) and presbyopia (18.6%; 95% CI, 5.2–37.7%). Cataract (7.4%), glaucoma (5.6%) and corneal scar or opacity (3.0%) were less prevalent. Age >50 years (OR = 11.96; 95% CI, 5.31–26.94) and a history of ocular trauma (OR = 5.21; 95% CI, 1.52–17.87) were associated with higher odds of ocular morbidity. Longer imprisonment was associated with xerophthalmia (OR = 20.1; 95% CI, 8.3–48.8%), whereas access to food from outside prison was associated with lower odds of ocular morbidity (OR = 0.45; 95% CI, 0.26–0.79).

**Conclusion:**

Ocular morbidity affects approximately four in ten prison inmates in SSA, with much of this burden preventable or treatable, despite considerable between-study heterogeneity and limited geographical evidence. Collectively, these findings support integrated prison eye care and standardized research to advance equitable interventions across SSA.

## Introduction

Prisons serve multiple functions, including retribution, deterrence, incapacitation, rehabilitation and reintegration (1). Although rehabilitation and reintegration seek to address factors that contribute to offending and support successful return to the community, incarceration itself can expose individuals to conditions that differ substantially from those experienced by free-living populations, including restricted healthcare access, overcrowding, poor sanitation and limited opportunities for preventive care (1–6). These conditions may increase vulnerability to ocular morbidity (7–9), while pre-existing or untreated eye diseases may further compromise education, vocational training, rehabilitation, and reintegration (1). Thus, the relationship between incarceration and eye health may be bidirectional, with the conditions of imprisonment influencing ocular health and ocular disease, which, in turn, can constrain successful rehabilitation and reintegration (1, 7–9).

Globally, an estimated 11.7 million people are incarcerated, more than one-third of whom are held in pretrial detention (6). Of this population, approximately 1.39 million people are incarcerated in Africa, including 992,000 in SSA (6), which has a regional imprisonment rate of 82 prisoners per 100,000 residents (10). Although men are substantially more likely than women to be incarcerated, women may face a disproportionate risk of pretrial detention (6). Across the region, persistent overcrowding has resulted in prison populations reaching approximately 150% of their intended capacity, further constraining access to adequate living conditions and essential health services (6). These conditions often coexist with poor ventilation, inadequate sanitation, insufficient nutrition and limited healthcare infrastructure (6), exposing incarcerated populations to a disproportionate burden of infectious, non-communicable and mental health conditions (11–13). For instance, the prevalence of human immunodeficiency virus (HIV) infection has been reported to range from 2.3% to 34.9%, whereas tuberculosis prevalence ranges from 0.4% to 16.4% in SSA prisons (13). Both conditions can have ocular manifestations such as conjunctival inflammation, cataract, uveitis, glaucoma, retinal disease, and other sight-threatening complications (14, 15). Furthermore, non-communicable diseases, particularly cardiovascular disease, remain prevalent in correctional facilities across SSA (11) and may increase susceptibility to ocular morbidity, including cortical and posterior subcapsular cataract (16).

Moreover, SSA prisons are characterized by high levels of violence that create unsafe environments and may increase the risk of ocular trauma, including corneal lacerations that can result in corneal scarring or opacity (6, 7, 17). Prison populations in SSA also face substantial food insecurity, with many inmates reliant on family food support (18, 19). Dietary assessments indicate inadequate intake of energy and fat, and limited access to polyunsaturated fatty acids and iron, underscoring persistent nutritional inadequacy in prison settings (20). Limited access to safe water, sanitation, hygiene supplies and healthcare, including hospital referral, preventive screening, health information and essential medicines, further compounds these vulnerabilities (18). Taken together, these environmental, nutritional and healthcare constraints may increase susceptibility to ocular morbidity, delay diagnosis and treatment, and exacerbate preventable visual impairment among incarcerated populations.

Of note, systemic differences between correctional facilities and free-living environments may influence the prevalence and spectrum of ocular morbidity and the factors that predispose inmates to these conditions. These differences may limit the utility of estimates derived from community-based populations for understanding ocular health within prisons and underscore the need for context-specific evidence. Such evidence is particularly important for identifying the ocular conditions that contribute most to visual morbidity in prison settings, recognizing modifiable risk factors, and informing the allocation of limited healthcare resources towards screening, treatment and prevention. Despite this need, there remains a lack of systematic synthesis of the prevalence and spectrum of eye diseases and their associated factors among prisoners in SSA. To fill this gap, this study conducted a systematic review and meta-analysis to estimate the prevalence and spectrum of ocular diseases and identify factors associated with ocular morbidity among incarcerated populations in SSA.

## Methods

This review followed a published protocol (21) developed in accordance with the Preferred Reporting Items for Systematic Review and Meta-Analysis Protocols (PRISMA-P) guidelines (22). The protocol specified the review objectives, eligibility criteria, search strategy, data extraction, risk-of-bias assessment and statistical methods, while reporting followed the PRISMA 2020 guidelines (23).

### Inclusion and exclusion criteria

Studies investigating the prevalence, distribution, associated risk factors, and/or diagnostic approaches for ocular disease among prison inmates in SSA were eligible for inclusion. Eligible studies used observational designs, including cross-sectional, retrospective comparative, and case–control studies; were conducted within correctional or detention facilities in SSA countries; and reported ocular examination findings using defined case ascertainment methods, including visual acuity assessment, slit-lamp examination, tonometry, ophthalmoscopy, or nutritional assessments such as body mass index (BMI), mid-upper arm circumference, or measurement of vitamin levels. Studies were required to report prevalence estimates or provide sufficient information to calculate prevalence. Studies were excluded if they were conducted outside SSA, included only non-incarcerated populations, were case reports, case series, narrative or systematic reviews, editorials, conference abstracts without extractable data, or were not available in English.

### Information sources and search strategy

A comprehensive literature search was performed in PubMed, Scopus, Web of Science (Core Collection), African Journals Online (AJOL), Embase, CINAHL (via EBSCO), APA PsycINFO (via EBSCO) and Google Scholar from database inception to March 2026 and updated the search in September, 2026. Search terms were developed around four core concepts: ocular disease, prevalence, prison populations, and geographical location. Keywords and controlled vocabulary terms, including variations of “prevalence,” “eye disease,” “ocular disorder,” “prison,” “incarcerated,” and “Sub-Saharan Africa,” were combined using Boolean operators (“AND” and “OR”), supplemented by individual country names within SSA (See supplementary S1). Reference lists of included studies and relevant reviews were manually screened to identify additional eligible records.

### Study selection and data extraction

Titles and abstracts were independently screened by two reviewers against predefined eligibility criteria. Cohen's kappa was used to assess the inter-rater agreement. Full-text assessment and data extraction were performed independently by the same reviewers, with disagreements resolved through discussion or consultation with a third reviewer when required. A standardized and piloted extraction form was used to collect information on study characteristics (author, year, design), setting (country, facility type, and security level where available), participant characteristics (sample size, sex distribution, age, and duration of incarceration), ocular disease outcomes, condition-specific prevalence estimates, associated risk factors and effect estimates, and diagnostic methods used for ocular disease assessment (see supplementary S2).

### Quality assessment and risk of bias

Risk of bias was assessed using the JBI Critical Appraisal Checklist for Studies Reporting Prevalence Data. Domain-level judgments (Yes/No/Unclear) were recorded for each item. Each criterion was scored as 1 when fulfilled (“Yes”) and 0 when rated as “No” or “Unclear.” Overall quality scores were categorized as low (≤49%), moderate (50%–69%), or high (≥70%). Traffic light plot was used to visualized each domain and summary plot to ascertain the overall evidence. The quality of included studies was also ascertained using the Quality Assessment Tool for Observational Cohort and Cross-sectional Studies. Quality assessment and risk of bias was conducted independently by two reviewers, with disagreements resolved through consensus. Inter-rater agreement was assessed using Cohen's kappa.

### Statistical analysis

A random-effects meta-analysis was performed to estimate the pooled prevalence of ocular disease across studies. Proportions were transformed using the Freeman–Tukey double arcsine transformation to stabilize variance before pooling, and estimates were subsequently back-transformed and presented with 95% confidence intervals (CIs) calculated using the Hartung-Knapp-Sidik-Jonkman (HK) adjustment. In addition, a 95% prediction interval (PI) was calculated alongside the pooled estimate to reflect the expected range of prevalence in future studies. Between-study variance (*τ*^2^) was estimated using the DerSimonian-Laird estimator. Subgroup analyses were conducted by individual ocular conditions to estimate condition-specific pooled prevalence. Between-study heterogeneity was evaluated using Cochran's Q statistic and quantified using the *I*^2^ statistic, with values interpreted as low (<24%), moderate (25%–49%), substantial (50%–74%), and high (≥75%) heterogeneity. Meta-analyses and forest plot generation were performed using R software version 4.5.0 via the metaprop function in the meta package. Sensitivity analyses were conducted to evaluate the influence of analytical choices on the pooled prevalence estimate. The primary analysis was repeated using a logit transformation as an alternative variance-stabilizing approach. In addition, a leave-one-out analysis was performed by sequentially excluding each included study and recalculating the pooled estimate to assess the influence of individual studies on the overall findings.

### Certainty of evidence (GRADE)

The certainty of the evidence for each important outcome was assessed using the GRADE (Grading of Recommendations Assessment, Development and Evaluation) approach. Because all included studies were observational, the certainty of evidence was initially rated as low and was further assessed across the domains of risk of bias, inconsistency, indirectness, and imprecision. The certainty of the evidence was evaluated using GRADEPro GDT software.

## Results

### Study selection and characteristics

The database search identified 977 records. After removal of 37 duplicate records, 940 records remained for title and abstract screening. Title and abstract screening showed substantial inter-rater agreement between the two independent reviewers (Cohen's *κ* = 0.70). Of these, 931 records were excluded as they did not meet the eligibility criteria, leaving 9 records assessed for full-text eligibility. Of the studies screened, six met the eligibility criteria and were included in the final analysis (Figure 1). The studies were conducted across four countries, with Nigeria contributing the largest number of studies (*n* = 3), followed by Tanzania, Uganda, and Kenya (*n* = 1 each) see Figure 2. Most studies used cross-sectional designs (*n* = 5), while one employed a retrospective comparative design. The total study population comprised approximately 3,361 participants, with individual sample sizes ranging from 152 to 1,048 participants. The included populations were predominantly male, with 2,515 males and 119 females reported across studies providing sex-specific data; one study included only male participants, and one retrospective comparative study did not report sex distribution. One study included a non-incarcerated comparison group (76 incarcerated and 76 non-incarcerated participants), while another incorporated a nested case–control component (247 cases and 448 controls) within a larger prison survey. Participant ages ranged from 9 to 83 years, with most studies focusing on adult incarcerated populations (see Table 1).

**Figure 1:**
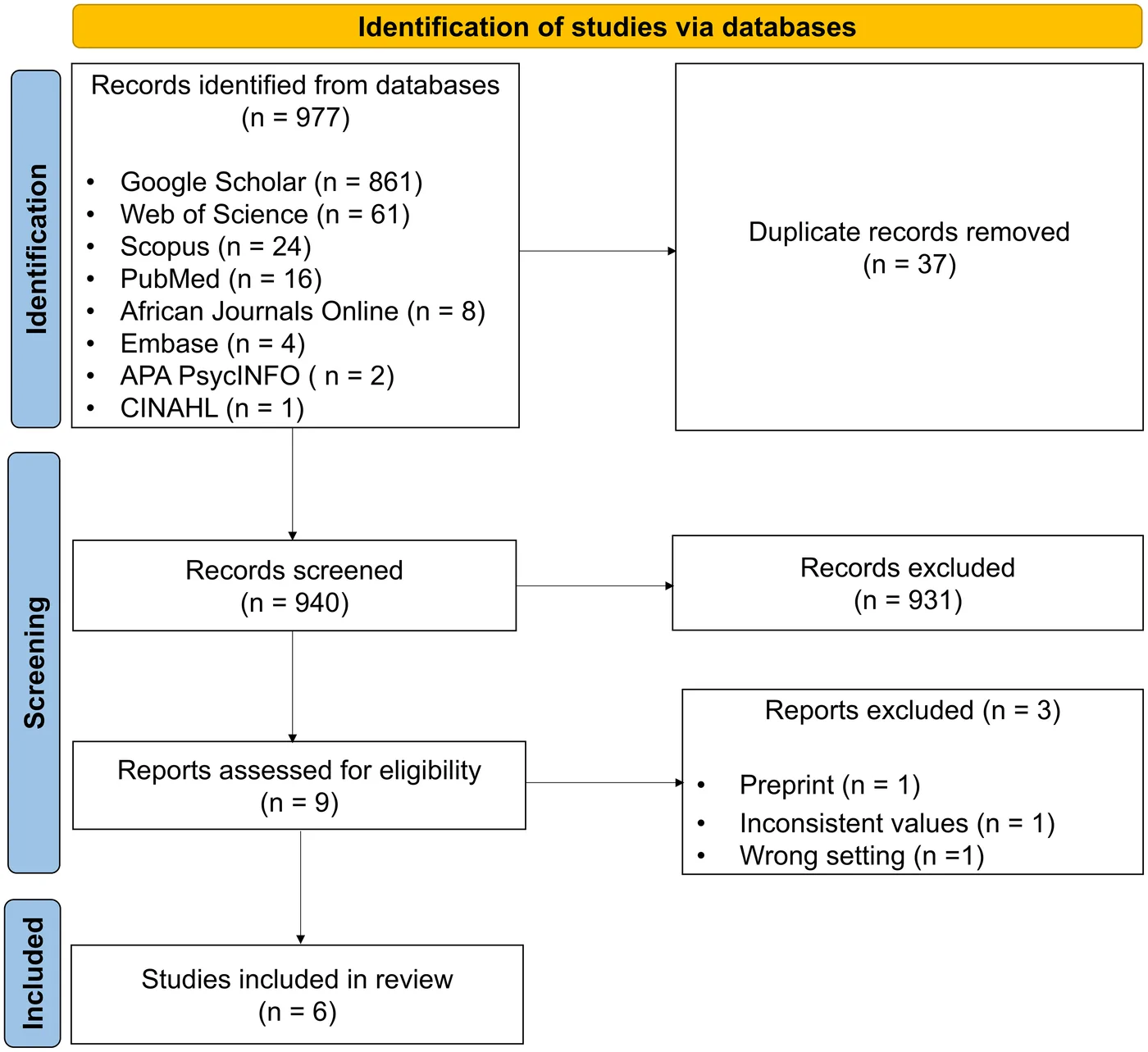
Study selection process.

**Figure 2:**
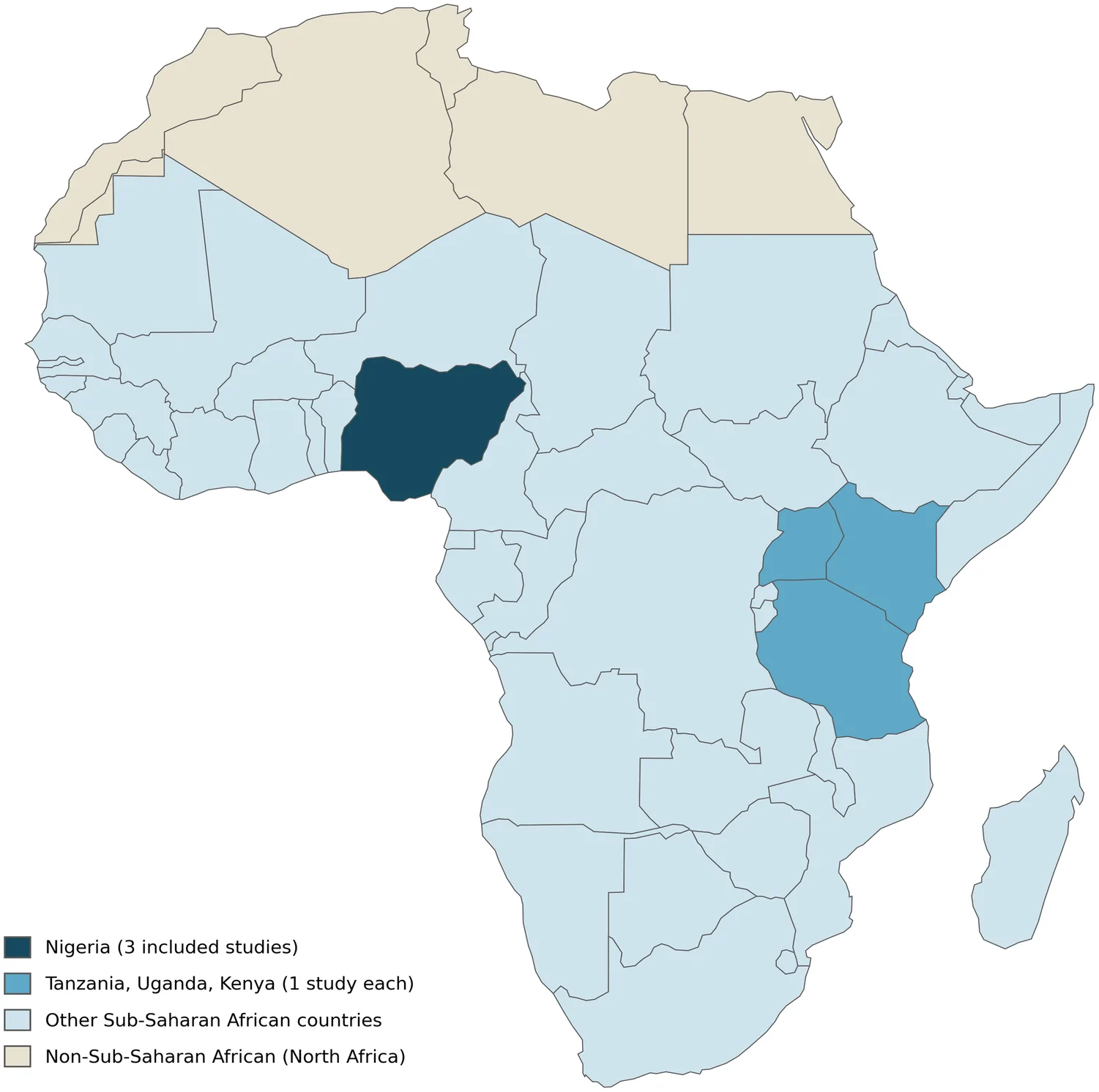
Geographical map of the evidence.

**Table 1:** Summary characteristics of included studies.

Author, Year	Country	Setting	Study design	Sample size (n)	Sex (M/F)	Age (years)
Mwakakonyole et al., 2024 (9)	Tanzania	Isanga Prison, Dodoma	Cross-sectional	274	M: 233	Median: 45 (range: 18–83)
					F: 41	
Zalwango et al., 2021 (7)	Uganda	Luzira Maximum Security Prison	Cross-sectional	367	M: 334	Mean: 39.1 ± 13.4 (range: 18–76)
					F: 33	
Ohikhuare, 2020 (24)	Nigeria	Oregun Juvenile Detention Centre	Retrospective comparative	152^†^	N/A	9–18
Ajite et al., 2011 (25)	Nigeria	Ilesa Medium Security Prison	Cross-sectional	515	M: 492	Mean: 32 ± 9.8 (range: 15–77)
					F: 23	
Ekwenchi et al., 2009 (26)	Nigeria	Onitsha Prison, Anambra State	Cross-sectional survey	1,005	M: 983	Mean: 27.3 (range: 16–73)
					F: 22	
Mathenge et al., 2007 (8)	Kenya	Nakuru Government Prison	Cross-sectional survey with nested case–control	1,048^‡^	M: 1,048	16 to >50

n, sample size; M, male; F, female.

† (76 I: 76 NI) I: incarcerated; NI: non-incarcerated.

‡ (247 cases/448 controls); N/A: Not available.

### Pooled prevalence of ocular morbidity

Figure 3 shows the pooled prevalence of ocular morbidity among prison inmates in SSA. The pooled prevalence was 43.1% (95% CI: 12.4–77.1%; 95% PI: 0.3–99.7%) based on 3,285 participants from six studies. Individual study estimates ranged from 8.0% to 88.2%, with considerable between-study heterogeneity (*I*^2^ = 99.2%, *τ*^2^ = 0.0613, *p* < 0.0001).

**Figure 3:**
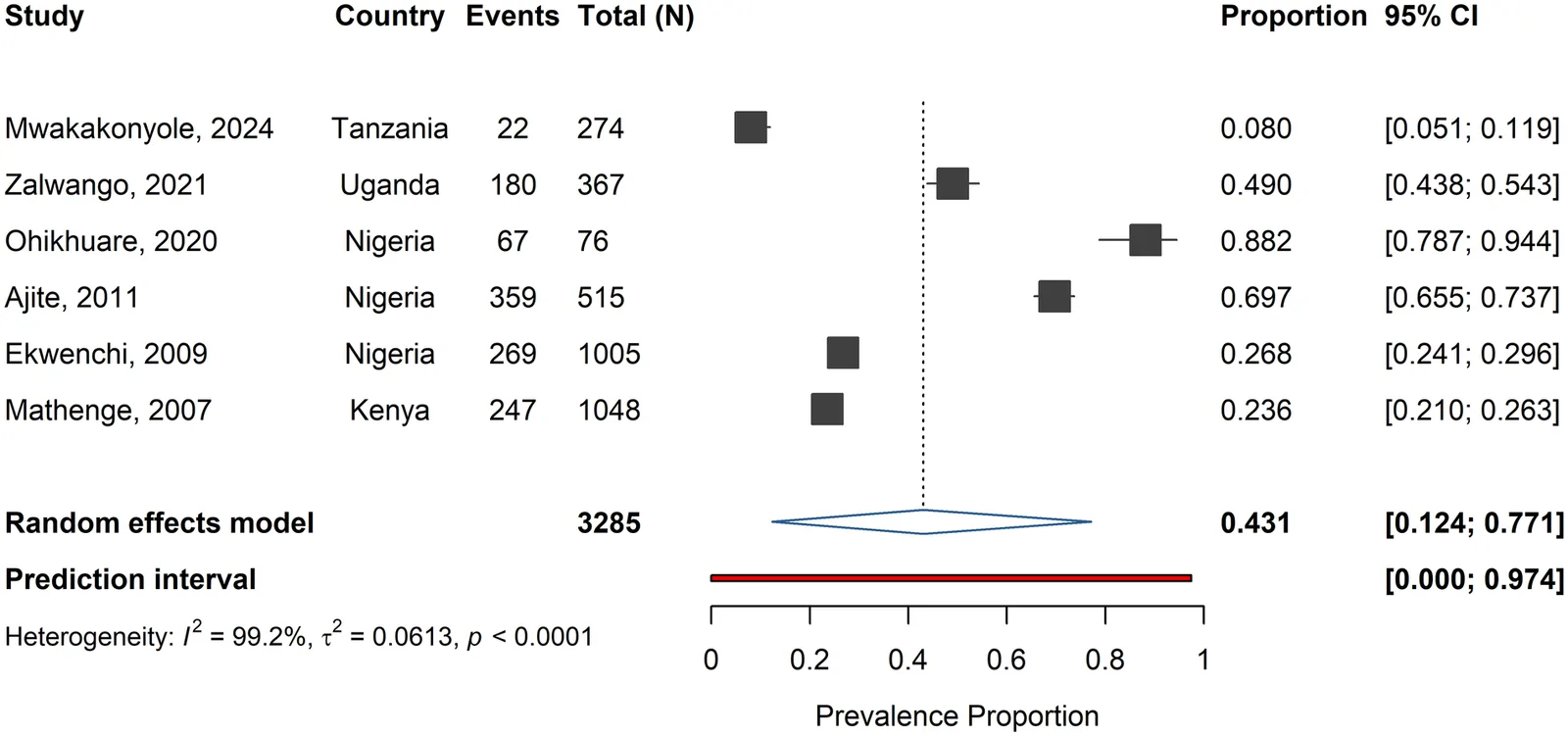
Forest plot of overall ocular disease prevalence among SSA prison inmates. The forest plot presents study-specific and pooled estimates of ocular disease prevalence among prison inmates across included studies. Individual study estimates are represented by squares and horizontal lines representing the corresponding 95% confidence intervals (CIs). The red horizontal bar below illustrates the 95% prediction interval The pooled prevalence estimate from the random-effects meta-analysis is represented by the diamond, with the centre indicating the pooled prevalence and the width representing the 95% CI. Proportions were transformed using the Freeman–Tukey double arcsine transformation to stabilize variance across studies. Between-study heterogeneity was assessed using the *I*^2^ statistic. Only incarcerated group prevalence estimate was used for Ohikhure 2020 study.

### Condition-specific meta-analyses

Meta-analyses by ocular condition showed variation in pooled prevalence estimates (see Table 2). Conjunctivitis had the highest pooled prevalence at 23.9% (95% CI: 10.7%–40.1%), followed by refractive error (19.5%**,** 95% CI: 12.5%–27.5%) and presbyopia (18.6%, 95% CI: 5.2%–37.7%). Posterior segment eye diseases and pterygium had pooled prevalences of 9.8% (95% CI: 2.5%–20.7%) and 9.3% (95% CI: 2.5%–19.4%), respectively, whereas cataract, glaucoma, and corneal scar/opacity were less common, with pooled prevalences of 7.4% (95% CI: 3.8%–11.8%), 5.6% (95% CI: 0.6%–14.2%), and 3.0% (95% CI: 1.3%–5.2%), respectively. High between-study heterogeneity was observed across all ocular conditions (*I*^2^ = 67.3%–98.7%), with the greatest heterogeneity for presbyopia (*I*^2^ = 98.7%) and conjunctivitis (*I*^2^ = 98.2%) (See Supplementary Figures in S3).

**Table 2:** Pooled prevalence estimates of ocular conditions among prison inmates.

Ocular condition	Included studies	Total sample size	Number of cases	Pooled prevalence, % (95% CI)	*I* ^2^	*τ* ^2^	*p*-value
Conjunctivitis	4	1,124	186	23.9 (10.7–40.1)	98.2	0.0303	<0.001
Refractive error	4	1,124	217	19.5 (12.5–27.5)	88.7	0.0079	<0.001
Presbyopia	3	1,048	166	18.6 (5.2–37.7)	98.7	0.0350	<0.001
Posterior segment eye diseases	4	793	59	9.8 (2.5–20.7)	91.5	0.0197	<0.001
Pterygium	3	1,048	107	9.3 (2.5–19.4)	95.3	0.0158	<0.001
Cataract	4	1,070	71	7.4 (3.8–11.8)	77.5	0.0038	0.004
Glaucoma	3	717	37	5.6 (0.6–14.2)	87.9	0.0003	0.003
Corneal scar/opacity	5	1,317	46	3.0 (1.3–5.2)	67.3	0.0157	0.016

Posterior segment diseases included optic atrophy, optic neuropathy, retinal detachment, maculopathy, diabetic retinopathy, hypertensive retinopathy, posterior uveitis, and retinitis pigmentosa.

### Sensitivity analyses

Two sensitivity analyses were performed. Using a logit transformation, the pooled prevalence was 41.8% (95% CI: 12.3%–78.6%; *I*^2^ = 98.9%), compared with 43.1% (95% CI: 12.4–77.1%; *I*^2^ = 99.2%) in the primary analysis. A leave-one-out sensitivity analysis was also conducted by sequentially excluding each of the six included studies. The pooled prevalence estimates ranged from 33.7% (95% CI: 7.6%–66.7%) to 51.6% (95% CI: 17.4%–84.9%), with the remaining estimates ranging from 37.4% to 47.4% (see supplementary S4). Excluding the study involving juveniles, which was also rated as having a high risk of bias, resulted in a pooled prevalence of 33.7% (95% CI: 7.6–66.7%), indicating that this study had a substantial influence on the overall pooled estimate.

### Distribution of ocular conditions across studies and countries

Figure 4 summarizes the distribution of reported ocular conditions across the included studies by country. Nigeria contributed the largest number of studies and reported the widest range of ocular conditions, including refractive error and presbyopia, glaucoma and glaucoma suspect, cataract and lens opacity, optic neuropathies, ocular surface diseases, corneal diseases, and other ocular conditions. Studies from Uganda reported refractive error and presbyopia, glaucoma and glaucoma suspect, cataract and lens opacity, optic neuropathies, ocular surface diseases, corneal diseases, vitamin A deficiency, and other ocular conditions. Tanzanian studies reported glaucoma and glaucoma suspects, cataract and lens opacity, optic neuropathies, and corneal diseases, whereas the Kenyan study revealed ocular surface diseases, corneal diseases, and vitamin A deficiency among prison inmates.

**Figure 4:**
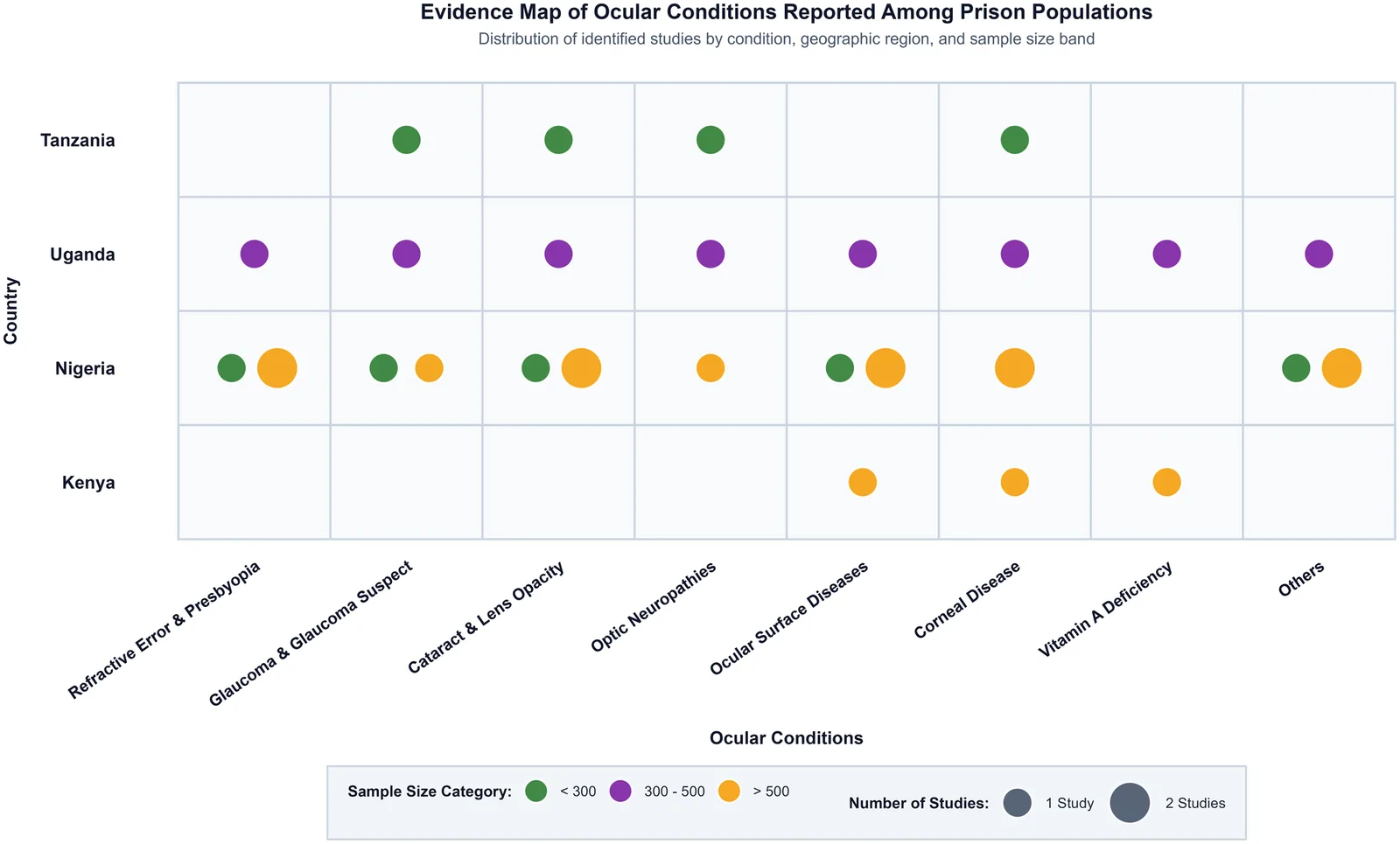
Presents an evidence map of ocular conditions reported among prison populations in SSA. The columns represent the reported ocular conditions, while rows indicate the countries of study origin. Circle size represent the number of studies that assessed each ocular condition, with circle colour corresponding to the sample size category (green: <300 participants; purple: 300–500 participants; orange: >500 participants).

### Risk factors associated with ocular diseases

Figure 5 presents individual-study estimates of factors associated with ocular disease. Older age (>50 years) showed a positive association with ocular diseases (adjusted OR: 11.96, 95% CI: 5.31–26.94), as did history of eye trauma (adjusted OR: 5.21, 95% CI: 1.52–17.87) (7). Longer duration of imprisonment was also associated positively with xerophthalmia (adjusted OR: 20.1, 95% CI: 8.3–48.8) (8). Non-prison food access was associated with lower odds of ocular diseases (adjusted OR: 0.45, 95% CI: 0.26–0.79) (7).

**Figure 5:**
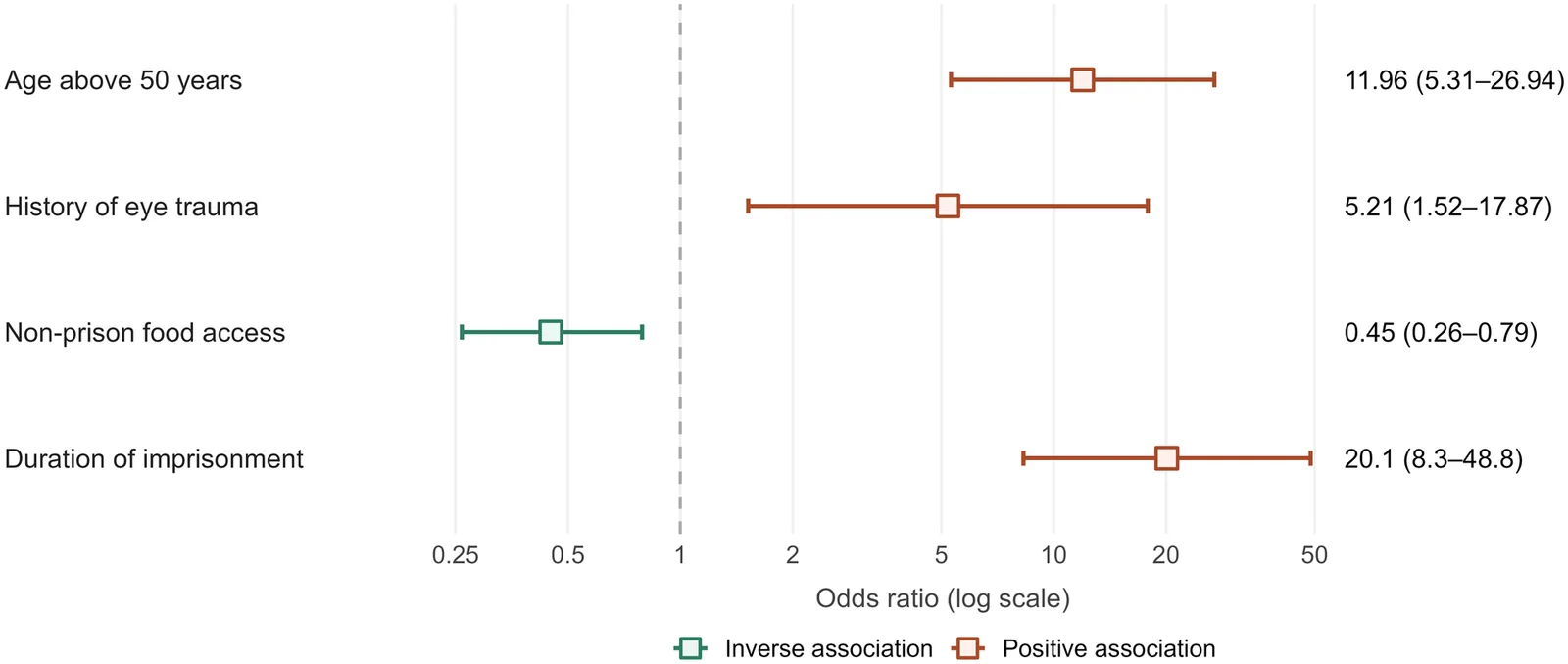
Association of risk factors with ocular diseases among included studies. Adjusted odds ratios (ORs) with 95% confidence intervals (CIs) from the included studies are presented on a logarithmic scale. Brown squares indicate risk-associated factors (OR > 1), and green squares indicate protective associations (OR < 1). The dashed vertical line represents the null value (OR = 1). Associations are presented for two included studies evaluating age >50 years, history of eye trauma, non-prison food access, and duration of imprisonment.

### Diagnostic tools used

The diagnostic approaches used to ascertain ocular disease varied considerably across studies (Table 3). All studies assessing adult populations employed Snellen-based visual acuity testing, generally supplemented with pinhole testing. Anterior segment and intraocular pressure assessment ranged from portable applanation and handheld tonometry with slit-lamp examination (7, 9, 25, 26) to simple magnifying-loupe examination without tonometry (8, 26). These assessments were not reported in the juvenile detention study (24). Posterior segment evaluation employed direct or indirect ophthalmoscopy in four studies and not reported in one. Only the Kenyan study incorporated systemic nutritional biomarkers (serum retinol, haemoglobin) and a validated night-blindness interview instrument. This heterogeneity in diagnostic depth and instrumentation represents a key methodological source of variability in reported prevalence estimates across studies.

**Table 3:** Diagnostic tools used in included studies.

Author, Year	Visual function examination	Anterior segment examination	Posterior segment examination	Other tools
Mwakakonyole et al., 2024 (9)	Snellen chart; Pinhole test	Icare tonometer; Slit lamp	Indirect ophthalmoscopy	Semi-structured questionnaire
Zalwango et al., 2021 (7)	Snellen chart (distance); Jaeger chart (near); Pinhole test	Perkins applanation tonometer; Portable slit lamp	Direct ophthalmoscopy	Questionnaire
Ajite et al., 2011 (25)	Snellen/illiterate “E” chart; Jaeger chart (near); Pinhole test	Perkins handheld tonometer; Pen torch	Direct/indirect ophthalmoscopy	Questionnaire
Ohikhuare, 2020 (24)	Snellen chart; Retinoscopy; Subjective refraction	Not reported	Direct ophthalmoscopy	Ishihara colour vision test
Mathenge et al., 2007 (8)	Not reported	Magnifying loupe (clinical exam)	Not reported	WHO 4-question night-blindness interview; Serum retinol (HPLC); Haemoglobin levels
Ekwenchi et al., 2009 (26)	Snellen chart	Torchlight/head loupe; Schiotz tonometer	Direct ophthalmoscopy	Questionnaire; Confrontation method (visual field)

### Quality assessment and risk of bias

Of the six included studies, five were rated as having overall low risk of bias (7, 9, 25, 26). These studies demonstrated appropriate sampling methods and frames, adequate sample sizes, clear descriptions of study populations and settings, adequate analytic coverage, valid and standardized condition assessment, appropriate statistical analyses, and adequate response rates. One study (24) was rated as having a high risk of bias with sampling frame, sample size, condition identification, and response rate rated as Unclear, and sampling method and measurement standardization rated as inadequate. Only study setting/subject description and analytic coverage were rated as adequate (See supplementary S5). At the item level, raw agreement was 83.3%, corresponding to a Cohen's kappa of 0.37, indicating fair agreement. Agreement on the overall methodological quality rating was higher, with a Cohen's kappa of 1.00.

In terms of quality assessment, three studies (7, 9, 26) were rated Good overall quality, with well-defined populations, adequate participation, consistent recruitment, valid exposure/outcome measures, and appropriate confounder adjustment; common limitations were cross-sectional temporality, no repeated exposure assessment, and unreported blinding. Two studies (8, 25) were rated Fair quality, sharing these limitations plus unreported sample size justification and no confounder adjustment. Lastly, one study (24) was rated Poor quality, with inconsistent sampling, no confounder adjustment, several unclear ratings for measurement validity (Q3, Q9, Q11), no sample size justification, and no examination of exposure levels (See supplementary S5).

### Overall certainty of evidence

The GRADE assessment of certainty of evidence indicated that the certainty for the pooled prevalence of ocular morbidity among incarcerated populations was very low. This assessment was driven primarily by the observational nature of the included studies, substantial unexplained between-study heterogeneity, and the small number and geographic concentration of contributing studies. Accordingly, the pooled prevalence estimate should be interpreted with appropriate caution (See supplementary S5).

## Discussion

This systematic review and meta-analysis characterized the burden, spectrum and associated factors of ocular morbidity among incarcerated populations in SSA. Across the limited and highly heterogeneous studies currently available from four sub-Saharan African countries, the pooled estimate suggests that approximately 43% of prison inmates had ocular morbidity involving the anterior, middle or posterior structures of the eye. Older age, ocular trauma and longer duration of imprisonment were associated with greater odds of ocular morbidity, whereas access to food from outside prison settings was associated with lower odds.

The global expansion of incarceration has placed increasing pressure on correctional systems, with many prisons operating beyond their intended capacity (6, 10). Overcrowding, inadequate infrastructure and constrained healthcare resources may create conditions that increase vulnerability to ocular disease through multiple pathways (6, 10). Poor sanitation and hygiene may increase exposure to infectious conditions with ocular manifestations (6, 13), while inadequate dietary intake may cause macro- and micronutrient deficiencies that compromise ocular health (18–20) and contribute to non-communicable conditions with ocular manifestations (11, 16). Limited access to preventive and specialist care may delay the detection and treatment of ocular conditions (18), whereas inadequate prison supervision and unsafe environments may increase exposure to violence and ocular trauma, with potential consequences that include corneal injury, scarring and visual impairment (6, 7, 17). These interrelated environmental, nutritional and healthcare constraints, together with individual factors, may therefore contribute to the burden of ocular morbidity among prisoners. We hypothesized that adverse prison conditions and individual characteristics would be associated with differences in the likelihood of ocular morbidity among incarcerated populations.

Approximately four in ten prison inmates had an ocular condition. To our knowledge, no previous systematic review has specifically assessed ocular disease prevalence in prison populations, limiting direct comparisons with other incarcerated populations; however, our findings are consistent with evidence of substantial unmet health needs, including ocular and nutritional disorders, in correctional settings. The pooled prevalence was higher than estimates reported in some SSA general populations (27–30) but lower than estimates reported among non-incarcerated populations in Ghana (35), Malawi (31) and Uganda (32). These differences should be interpreted cautiously because prison populations differ from community populations in demographic composition, socioeconomic circumstances, health profiles and access to healthcare, all of which may influence ocular morbidity (7, 33). Restricted access to preventive and specialist eye care within correctional facilities may also delay diagnosis and treatment (34, 35), while limited and inconsistently updated prison-based epidemiological data may affect the completeness and comparability of available estimates (7, 9, 25). Collectively, these findings underscore the need for context-specific evidence and prison-based eye health strategies that reflect the distinct demographic, environmental and healthcare conditions of incarcerated populations.

Condition-specific meta-analysis showed that conjunctivitis was the most prevalent ocular condition among prisoners, a finding that is biologically plausible given the environmental conditions common in correctional facilities, including overcrowding, poor ventilation, dust exposure and inadequate hygiene, which may increase exposure of the conjunctiva to allergens and microorganisms (6, 18, 36). Although conjunctivitis rarely causes permanent visual loss, persistent symptoms can impair quality of life and daily functioning (37). Refractive error was the second most prevalent condition (19.5%), substantially lower than the 48.61% reported in the general SSA population, but still indicative of a considerable burden of avoidable visual impairment (38). Unlike many ocular conditions, refractive error can often be corrected with spectacles (39), yet limited access to routine refraction and spectacle services within correctional facilities may leave affected inmates without appropriate correction. These findings support the integration of periodic vision and refractive screening into prison healthcare programmes as a feasible approach to reduce avoidable visual disability among incarcerated populations.

In this review, older age, longer duration of imprisonment, and previous ocular trauma were associated with higher odds of ocular morbidity in the two studies that examined these factors. For instance, older prisoners had substantially higher odds of ocular morbidity, with those aged over 50 years showing approximately 12-fold greater odds. This finding is consistent with the established epidemiology of age-related ocular conditions, including cataract, glaucoma and presbyopia, which increase with advancing age because of cumulative degenerative changes (40–42). Previous ocular trauma was also associated with higher odds of ocular morbidity (7), consistent with evidence linking ocular injury to corneal scarring (43), traumatic cataract (44), secondary glaucoma (45) and other vision-threatening complications (46). Longer imprisonment was associated with a markedly higher likelihood of xerophthalmia, with approximately 20-fold greater odds (8), which may reflect cumulative exposure to adverse prison conditions, including inadequate and nutritionally limited diets (8, 20). Prolonged nutritional inadequacy, particularly insufficient vitamin A intake, may compromise ocular surface health and contribute to xerophthalmia and other ocular conditions over time.

Receipt of food from outside correctional facilities was significantly associated with lower odds of ocular morbidity. Food obtained from outside prison may provide greater dietary diversity and improve access to nutrients that are often limited in institutional diets. Reports from correctional settings indicate that prison diets may be deficient in essential micronutrients (20). Although evidence on nutritional factors of ocular morbidity in prison populations remains limited, this lower odds association highlights nutrition as a potentially modifiable component of prison eye health. Where improvements in the diversity of prison diets are difficult to achieve, targeted micronutrient interventions such as vitamin A supplementation or fortification of commonly consumed foods could provide a practical approach to addressing nutritional deficiencies.

Notably, the current evidence base has a high underrepresentation of female incarcerated populations. Of the 3,361 participants across the six included studies, only 119 were female. This limits the extent to which the pooled findings can be considered representative of female prisoners, particularly because prison conditions, healthcare access and needs, and nutritional exposures, differ between male and female incarcerated populations (4, 47). Such differences may also have implications for ocular health, as female prisoners have been reported to experience a greater burden of certain chronic health conditions, including diabetes, hypertension, infectious diseases and genitourinary diseases, which may have ocular manifestations or contribute to long-term ocular morbidity (48–51). As the number of women in prison continues to increase (4, 47), future research should ensure adequate inclusion of female incarcerated populations and report ocular morbidity estimates stratified by sex to better characterize potential differences in disease burden and associated factors.

### Clinical and policy implications

An important clinical implication is the need for clear referral pathways linking prison health services with appropriate ophthalmic and hospital facilities. Such pathways could define criteria for routine and urgent referral, identify designated referral facilities, and establish procedures for arranging transport and communicating clinical information, particularly for prisoners with potentially sight-threatening conditions or ocular emergencies. Prison healthcare and custodial staff should also receive basic training to recognize ocular emergencies, including sudden visual loss, ocular trauma, and chemical injuries, and to facilitate prompt referral. Prison health systems should also strengthen nutrition programmes, injury-prevention measures, ophthalmic infrastructure, equipment, and healthcare personnel to address modifiable contributors to ocular morbidity. At the policy level, prison eye health should be incorporated into national eye health strategies and universal health coverage initiatives to ensure that incarcerated populations have equitable access to essential eyecare services.

### Strengths and limitations

This review has some strengths and limitations. To our knowledge, it is the first systematic reviews to synthesize the prevalence, spectrum and associated factors of ocular morbidity among incarcerated populations across SSA, addressing an understudied population in ophthalmic epidemiology. We used a comprehensive search strategy, standardized study selection and data extraction, formal quality appraisal, and meta-analysis to synthesize available evidence across prison settings. However, several limitations warrant consideration. The very high between-study heterogeneity limits the precision and generalizability of the pooled prevalence estimate, which should therefore be interpreted as an overall summary rather than a precise regional estimate. Only six studies from four countries met the inclusion criteria, leaving substantial geographic gaps and limiting representation of the diverse prison systems across SSA. Furthermore, the evidence base was disproportionately represented by Nigerian prison populations, which contributed the largest number of participants, and may therefore have exerted greater influence on the pooled estimate. In addition, one study was rated as having low methodological quality, and the predominantly cross-sectional evidence base limits assessment of temporal or causal relationships between incarceration-related exposures and ocular morbidity. Lastly, restriction of eligibility to studies published in English may have excluded relevant data from francophone SSA countries, introducing potential language bias. These limitations underscore the need for larger, multi-country studies with standardized sampling, diagnostic protocols and longitudinal designs to generate more comparable and generalizable evidence.

## Conclusion

This review demonstrates a substantial burden of ocular morbidity among incarcerated populations in SSA, with conjunctivitis, refractive error, and presbyopia among the most frequently reported conditions, while cataract, glaucoma, and posterior segment diseases, although less prevalent, represented an important burden of potentially sight-threatening pathology. Older age, ocular trauma, and longer duration of incarceration were associated with greater odds of ocular morbidity, whereas access to food outside prison was associated with lower odds, highlighting the potential contribution of nutritional and environmental factors to prison eye health. These findings underscore the need to integrate eye health into routine prison healthcare through comprehensive eye examinations at entry and periodic assessments thereafter, with access to refractive services, affordable spectacles, timely treatment, and clear referral pathways for specialist ophthalmic care. Future multi-country studies using standardized ophthalmic examination protocols and including both male and female incarcerated populations are needed to improve the representativeness of prevalence estimates, while longitudinal research should examine the incidence and progression of ocular disease and clarify the effects of nutritional status, duration of incarceration, healthcare access, trauma, and environmental conditions on ocular health.
