# Supplementary material for: Burden, spectrum and associated factors of ocular disease among incarcerated populations in sub-Saharan Africa: a systematic review and meta-analysis
Source: Front Med (Lausanne). 2026 Oct 23;13:1969779. doi: 10.3389/fmed.2026.1969779 (PMC13741224; doi:10.3389/fmed.2026.1969779)
Supplement: Supplementary file 1 [file Supplementaryfile1.docx]

**Search Strategies and Search Documentation**

**Review title:**

***Prevalence of Eye Diseases Among Prison Inmates in Sub-Saharan Africa: A Systematic Review***

**S1.1 Information sources and search approach**

The literature search was designed and conducted by the review team's information specialist/librarian in consultation with the review team. The search strategy combined three principal concepts: **prison/inmate populations**, **eye diseases and visual disorders**, and **Sub-Saharan Africa**.

The original searches were conducted on **21 March 2026**. The searches were subsequently **updated on 16 September 2026**, with additional databases to identify records newly indexed or published after the original search.

The databases and information sources searched were **PubMed, Scopus, Web of Science Core Collection, Google Scholar, African Journals Online (AJOL), Embase, APA PsycINFO and CINAHL**. Google Scholar was searched using **Publish or Perish (PoP), version 8**, because it permits systematic retrieval and recording of search results from Google Scholar.

Database-specific syntax was adapted to the functionality of each platform. Controlled vocabulary was combined with free-text terms in databases supporting subject headings. For platforms with more limited search functionality, including Google Scholar and AJOL, multiple simplified keyword searches were conducted.

**S1.2 PubMed**

**Database:** PubMed
**Platform:** PubMed, National Library of Medicine
**Original search date:** 21 March 2026
**Updated search date:** 16 September 2026
**Original records retrieved:** 14
**Updated records retrieved:** 16
**Limits/filters:** No publication-date, language, age, sex or study-design filter was applied at the database-search stage. The review's English-language eligibility restriction was applied during study selection.
**Search fields:** MeSH terms and Title/Abstract [tiab] fields.

**Controlled vocabulary**

The PubMed search incorporated the following MeSH concepts:

- **Prisoners [MeSH]**
- **Eye Diseases [MeSH]**
- **Vision Disorders [MeSH]**
- **Africa [MeSH]**
- **Africa South of the Sahara [MeSH]**

**Free-text concepts**

Free-text terms covered:

**Prison population:**
prison*, prisoner*, inmate*, jail*, gaol*, incarcerat*, detainee*, detain*, detention, custod*, correctional facilit*, correctional institution*, correctional service*, penal institution*, penitentiary, remand, convict*, offender*, prison population*

**Eye/ocular conditions:**

eye disease*, ocular disease*, ophthalmic disease*, ocular morbid*, eye disorder*, ocular disorder*, visual impair*, vision impair*, low vision, blindness, cataract*, glaucoma*, conjunctivit*, trachoma*, pterygi*, keratit*, corneal opacit*, uveitis, retinopath*, maculopath*, refractive error*, myopi*, hyperopi*, presbyopi*, astigmati*

**Geography:**
Africa, Sub-Saharan Africa, Sub-Saharan African, Africa South of the Sahara, West Africa, East Africa, Central Africa, Southern Africa, and individual Sub-Saharan African countries.

**Final PubMed search strategy**

(Prisoners[Mesh] OR prison*[tiab] OR prisoner*[tiab] OR inmate*[tiab] OR jail*[tiab] OR gaol*[tiab] OR incarcerat*[tiab] OR detainee*[tiab] OR detain*[tiab] OR detention[tiab] OR custod*[tiab] OR "correctional facilit*"[tiab] OR "correctional institution*"[tiab] OR "correctional service*"[tiab] OR "penal institution*"[tiab] OR penitentiary[tiab] OR remand[tiab] OR convict*[tiab] OR offender*[tiab] OR "prison population*"[tiab])

AND

("Eye Diseases"[Mesh] OR "Vision Disorders"[Mesh] OR "eye disease*"[tiab] OR "ocular disease*"[tiab] OR"ophthalmic disease*"[tiab] OR "ocular morbid*"[tiab] OR "eye disorder*"[tiab] OR "ocular disorder*"[tiab] OR "visual impair*"[tiab] OR "vision impair*"[tiab] OR "low vision"[tiab] OR blindness[tiab] OR cataract*[tiab] OR glaucoma*[tiab] OR conjunctivit*[tiab] OR trachoma*[tiab] OR pterygi*[tiab] OR keratit*[tiab] OR "corneal opacit*"[tiab] OR uveitis[tiab] OR retinopath*[tiab] OR maculopath*[tiab] OR "refractive error*"[tiab] OR myopi*[tiab] OR hyperopi*[tiab] OR presbyopi*[tiab] OR astigmati*[tiab]) OR (presbyopi*[Title/Abstract])) OR cataract*[ tiab] OR glaucoma*[ tiab] OR conjunctivit*[tiab] OR trachoma*[ tiab] OR (pterygi*[ tiab] OR (keratit*[ tiab] OR ("corneal opacit*"[ tiab] OR (uveitis[tiab] OR (retinopath*[ tiab] OR "diabetic retinopathy"[ tiab] OR "retinal disease*"[ tiab] OR "optic neuropath*"[ tiab] OR maculopath*[ tiab] OR "eye infection*"[ tiab] OR ophthalmolog*[ tiab] OR "eye health"[ tiab] OR "ocular health"[ tiab]

AND

(Africa[Mesh] OR "Africa South of the Sahara"[Mesh] OR Africa[tiab] OR "sub-Saharan Africa"[tiab] OR "Sub-Saharan African"[tiab] OR "West Africa"[tiab] OR "East Africa"[tiab] OR "Central Africa"[tiab] OR "Southern Africa"[tiab] OR Angola[tiab] OR Benin[tiab] OR Botswana[tiab] OR "Burkina Faso"[tiab] OR Burundi[tiab] OR "Cabo Verde"[tiab] OR "Cape Verde"[tiab] OR Cameroon[tiab] OR "Central African Republic"[tiab] OR Chad[tiab] OR Comoros[tiab] OR "Republic of the Congo"[tiab] OR "Democratic Republic of the Congo"[tiab] OR Djibouti[tiab] OR "Equatorial Guinea"[tiab] OR Eritrea[tiab] OR Eswatini[tiab] OR Ethiopia[tiab] OR Gabon[tiab] OR Gambia[tiab] OR Ghana[tiab] OR Guinea[tiab] OR "Guinea-Bissau"[tiab] OR Kenya[tiab] OR Lesotho[tiab] OR Liberia[tiab] OR Madagascar[tiab] OR Malawi[tiab] OR Mali[tiab] OR Mauritania[tiab] OR Mauritius[tiab] OR Mozambique[tiab] OR Namibia[tiab] OR Niger[tiab] OR Nigeria[tiab] OR Rwanda[tiab] OR "Sao Tome and Principe"[tiab] OR Senegal[tiab] OR Seychelles[tiab] OR "Sierra Leone"[tiab] OR Somalia[tiab] OR "South Africa"[tiab] OR "South Sudan"[tiab] OR Tanzania[tiab] OR Togo[tiab] OR Uganda[tiab] OR Zambia[tiab] OR Zimbabwe[tiab] OR "Cote d'Ivoire"[tiab] OR "Côte d'Ivoire"[tiab] OR "Ivory Coast"[tiab])

**Search results:** 21 March 2026 = **14 records**; 16 September 2026 = **16 records**.

**S1.3 Scopus**

**Database:** Scopus
**Platform:** Elsevier Scopus
**Original search date:** 21 March 2026
**Updated search date:** 16 September 2026
**Original records retrieved:** 20
**Updated records retrieved:** 24
**Limits/filters:** No additional publication-date, language or study-design filter applied at the database-search stage.
**Search fields:** TITLE-ABS-KEY.

**Controlled vocabulary**

Scopus does not provide a MeSH-style controlled vocabulary equivalent to PubMed. The search therefore relied on database-indexed text fields using free-text terms.

**Free-text concepts**

The search incorporated synonyms for:

- prison/inmate populations;
- eye and ocular diseases;
- visual impairment and blindness;
- specific ophthalmic conditions; and
- Sub-Saharan African countries and regional terminology.

**Final Scopus search strategy**

TITLE-ABS-KEY( prison* OR prisoner* OR inmate* OR jail* OR gaol* OR incarcerat* OR detainee* OR detain* OR detention OR custod* OR "correctional facilit*" OR "correctional institution*" OR "correctional service*" OR "penal institution*" OR penitentiary OR penitentiaries OR remand OR convict* OR offender* OR "prison population*" OR "incarcerated person*" OR "incarcerated people" )

AND

TITLE-ABS-KEY( "eye disease*" OR "ocular disease*" OR "ophthalmic disease*" OR "ocular morbid*" OR "eye disorder*" OR "ocular disorder*" OR "ophthalmic disorder*" OR "visual impair*" OR "vision impair*" OR "low vision" OR blindness OR blind OR "eye condition*" OR "ocular condition*" OR "refractive error*" OR ametropi* OR myopi* OR hyperopi* OR hypermetropi* OR astigmati* OR presbyopi* OR cataract* OR glaucoma* OR conjunctivit* OR trachoma* OR pterygi* OR keratit* OR "corneal opacit*" OR uveitis OR retinopath* OR "diabetic retinopathy" OR "retinal disease*" OR "optic neuropath*" OR maculopath* OR "eye infection*" OR ophthalmolog* OR "eye health" OR "ocular health" )

AND

TITLE-ABS-KEY( "Sub-Saharan Africa" OR "sub Saharan Africa" OR "West Africa" OR "East Africa" OR "Central Africa" OR "Southern Africa" OR Angola OR Benin OR Botswana OR "Burkina Faso" OR Burundi OR "Cabo Verde" OR "Cape Verde" OR Cameroon OR "Central African Republic" OR Chad OR Comoros OR "Republic of the Congo" OR "Congo Brazzaville" OR "Democratic Republic of the Congo" OR DRC OR "Djibouti" OR "Equatorial Guinea" OR Eritrea OR Eswatini OR Swaziland OR Ethiopia OR Gabon OR Gambia OR Ghana OR Guinea OR "Guinea-Bissau" OR Liberia OR Kenya OR Lesotho OR Madagascar OR Malawi OR Mali OR Mauritania OR Mauritius OR Mozambique OR Namibia OR Niger OR Nigeria OR Rwanda OR "Sao Tome and Principe" OR Senegal OR Seychelles OR "Sierra Leone" OR Somalia OR "South Africa" OR "South Sudan" OR Tanzania OR Togo OR Uganda OR Zambia OR Zimbabwe OR "Cote d'Ivoire" OR "Côte d'Ivoire" OR "Ivory Coast" )

**Search results:** 21 March 2026 = **20 records**; 16 September 2026 = **24 records**.

**S1.4 Web of Science Core Collection**

**Database:** Web of Science Core Collection
**Platform:** Clarivate Web of Science
**Original search date:** 21 March 2026
**Updated search date:** 16 September 2026
**Original records retrieved:** 58
**Updated records retrieved:** 61
**Limits/filters:** No additional publication-date, language or study-design filter applied at the database-search stage.
**Search field:** Topic (TS).

**Controlled vocabulary**

Web of Science Core Collection does not use a controlled vocabulary equivalent to MeSH. The strategy therefore used free-text Topic searching.

**Final Web of Science search strategy**

TS=( prison* OR prisoner* OR inmate* OR jail* OR gaol* OR incarcerat* OR detainee* OR detain* OR detention OR custod* OR "correctional facilit*" OR "correctional institution*" OR "correctional service*" OR "penal institution*" OR penitentiary OR penitentiaries OR remand OR convict* OR offender* OR "prison population*" OR "incarcerated person*" OR "incarcerated people" )

AND

TS=( "eye disease*" OR "ocular disease*" OR "ophthalmic disease*" OR "ocular morbid*" OR "eye disorder*" OR "ocular disorder*" OR "ophthalmic disorder*" OR "visual impair*" OR "vision impair*" OR "low vision" OR blindness OR blind OR "eye condition*" OR "ocular condition*" OR "refractive error*" OR ametropi* OR myopi* OR hyperopi* OR hypermetropi* OR astigmati* OR presbyopi* OR cataract* OR glaucoma* OR conjunctivit* OR trachoma* OR pterygi* OR keratit* OR "corneal opacit*" OR uveitis OR retinopath* OR "diabetic retinopathy" OR "retinal disease*" OR "optic neuropath*" OR maculopath* OR "eye infection*" OR ophthalmolog* OR "eye health" OR "ocular health" )

AND

TS= ("Sub-Saharan Africa" OR "sub saharan Africa" OR "West Africa" OR "East Africa" OR "Central Africa" OR "Southern Africa" OR Angola OR Benin OR Botswana OR "Burkina Faso" OR Burundi OR "Cabo Verde" OR "Cape Verde" OR Cameroon OR "Central African Republic" OR Chad OR Comoros OR "Republic of the Congo" OR "Congo Brazzaville" OR "Democratic Republic of the Congo" OR DRC OR "Congo Kinshasa" OR Djibouti OR "Equatorial Guinea" OR Eritrea OR Eswatini OR Swaziland OR Ethiopia OR Gabon OR Gambia OR Ghana OR Guinea OR "Guinea-Bissau" OR Liberia OR Kenya OR Lesotho OR Madagascar OR Malawi OR Mali OR Mauritania OR Mauritius OR Mozambique OR Namibia OR Niger OR Nigeria OR Rwanda OR "Sao Tome and Principe" OR Senegal OR Seychelles OR "Sierra Leone" OR Somalia OR "South Africa" OR "South Sudan" OR Tanzania OR Togo OR Uganda OR Zambia OR Zimbabwe OR "Cote d'Ivoire" OR "Côte d'Ivoire" OR "Ivory Coast" )

**Search results:** 21 March 2026 = **58 records**; 16 September 2026 = **61 records**

**S1.6 Google Scholar**

**Database/source:** Google Scholar
**Search interface:** Google Scholar accessed through Publish or Perish (PoP), version 8
**Original search date:** 21 March 2026
**Updated search date:** 16 September 2026

Google Scholar was searched using five separate simplified keyword searches. The searches were conducted separately because Google Scholar does not provide the same reproducible database-search functionality as structured bibliographic databases.

Results were retrieved in relevance order using Publish or Perish. A maximum of **200 records per search** was specified. The records retrieved from the individual searches were subsequently combined, and overlapping records were identified and removed before integration into the review dataset.

**Google Scholar search set**

| **Search** | **Exact query** | **Original results** | **Updated results** |
| --- | --- | --- | --- |
| GS-1 | prisoners "eye disease" Africa | [200] | [200] |
| GS-2 | prison inmates "visual impairment" Africa | [200] | [200] |
| GS-3 | incarcerated blindness Africa | [200] | [200] |
| GS-4 | prison cataract glaucoma Africa | [200] | [200] |
| GS-5 | "refractive error" prisoners Africa | [61] | [61] |
| **Total** |  | **861*** | **861*** |

*The original Google Scholar total of 861 represents the number of records retrieved/captured across the five searches after accounting for the actual retrieval output; it should not be interpreted as five independent sets of 200 unique records.

**ADDITIONAL DATABASES ADDED**

**S1.7 African Journals Online**

**Database/source:** African Journals Online (AJOL)
**Search interface:** African Journal Online
**Search date:** 16 September 2026

AJOL was searched using five separate simplified keyword searches. The searches were conducted separately because the platform has limited support for advanced Boolean operators and complex search strings and does not support bulk export. The **first 10 pages** were screened and potentially relevant papers were exported for screening.

| **Search** | **Exact query** | **Results** |
| --- | --- | --- |
| S1 | prisoners "eye disease" Africa | 2 relevant papers |
| S2 | 2 relevant papers | 2 relevant papers |
| S3 | incarcerated blindness Africa | 2 relevant papers |
| S4 | prison cataract glaucoma Africa | 2 relevant papers |
| S5 | "refractive error" prisoners Africa | No relevant paper |
| **Total** |  | **8** |

**S1.7 Embase**

**Database:** Embase
**Platform:** Elsevier Embase
**Search date:** 17 September 2026
**Records retrieved:** 4
**Limits/filters:** No additional publication-date, language or study-design filter applied at the database-search stage.
**Search field:** ti,ab,kw.

**Controlled vocabulary**

Emabse does not provide a MeSH-style controlled vocabulary equivalent to PubMed. The search therefore relied on database-indexed text fields using free-text terms.

**Final Embase search strategy**

prison*:ti,ab,kw OR prisoner*:ti,ab,kw OR inmate*:ti,ab,kw OR jail*:ti,ab,kw OR gaol*:ti,ab,kw OR incarcerat*:ti,ab,kw OR detainee*:ti,ab,kw OR detain*:ti,ab,kw OR detention:ti,ab,kw OR custod*:ti,ab,kw OR 'correctional facilit*':ti,ab,kw OR 'correctional institution*':ti,ab,kw OR 'correctional service*':ti,ab,kw OR 'penal institution*':ti,ab,kw OR penitentiary:ti,ab,kw OR penitentiaries:ti,ab,kw OR remand:ti,ab,kw OR convict*:ti,ab,kw OR offender*:ti,ab,kw OR 'prison population*':ti,ab,kw OR 'incarcerated person*':ti,ab,kw OR 'incarcerated people':ti,ab,kw

AND

'eye disease*':ti,ab,kw OR 'ocular disease*':ti,ab,kw OR 'ophthalmic disease*':ti,ab,kw OR 'ocular morbid*':ti,ab,kw OR 'eye disorder*':ti,ab,kw OR 'ocular disorder*':ti,ab,kw OR 'ophthalmic disorder*':ti,ab,kw OR 'visual impair*':ti,ab,kw OR 'vision impair*':ti,ab,kw OR 'low vision':ti,ab,kw OR blindness:ti,ab,kw OR blind:ti,ab,kw OR 'eye condition*':ti,ab,kw OR 'ocular condition*':ti,ab,kw OR 'refractive error*':ti,ab,kw OR ametropi*:ti,ab,kw OR myopi*:ti,ab,kw OR hyperopi*:ti,ab,kw OR hypermetropi*:ti,ab,kw OR astigmati*:ti,ab,kw OR presbyopi*:ti,ab,kw OR cataract*:ti,ab,kw OR glaucoma*:ti,ab,kw OR conjunctivit*:ti,ab,kw OR trachoma*:ti,ab,kw OR pterygi*:ti,ab,kw OR keratit*:ti,ab,kw OR 'corneal opacit*':ti,ab,kw OR uveitis:ti,ab,kw OR retinopath*:ti,ab,kw OR 'diabetic retinopathy':ti,ab,kw OR 'retinal disease*':ti,ab,kw OR 'optic neuropath*':ti,ab,kw OR maculopath*:ti,ab,kw OR 'eye infection*':ti,ab,kw OR ophthalmolog*:ti,ab,kw OR 'eye health':ti,ab,kw OR 'ocular health':ti,ab,kw

AND

('africa south of the sahara':ti,ab,kw OR 'sub-saharan africa':ti,ab,kw OR ssa:ti,ab,kw OR 'west africa':ti,ab,kw OR 'east africa':ti,ab,kw OR 'central africa':ti,ab,kw OR 'southern africa':ti,ab,kw OR angola:ti,ab,kw OR benin:ti,ab,kw OR botswana:ti,ab,kw OR 'burkina faso':ti,ab,kw OR burundi:ti,ab,kw OR 'cabo verde':ti,ab,kw OR 'cape verde':ti,ab,kw OR cameroon:ti,ab,kw OR 'central african republic':ti,ab,kw OR chad:ti,ab,kw OR comoros:ti,ab,kw OR 'republic of the congo':ti,ab,kw OR 'congo brazzaville':ti,ab,kw OR 'democratic republic of the congo':ti,ab,kw OR drc:ti,ab,kw OR 'congo kinshasa':ti,ab,kw OR djibouti:ti,ab,kw OR 'equatorial guinea':ti,ab,kw OR eritrea:ti,ab,kw OR eswatini:ti,ab,kw OR ethiopia:ti,ab,kw OR gabon:ti,ab,kw OR gambia:ti,ab,kw OR ghana:ti,ab,kw OR guinea:ti,ab,kw OR 'guinea-bissau':ti,ab,kw OR liberia:ti,ab,kw OR kenya:ti,ab,kw OR lesotho:ti,ab,kw OR madagascar:ti,ab,kw OR malawi:ti,ab,kw OR mali:ti,ab,kw OR mauritania:ti,ab,kw OR mauritius:ti,ab,kw OR mozambique:ti,ab,kw OR namibia:ti,ab,kw OR niger:ti,ab,kw OR nigeria:ti,ab,kw OR rwanda:ti,ab,kw OR 'sao tome':ti,ab,kw) AND principe:ti,ab,kw OR senegal:ti,ab,kw OR seychelles:ti,ab,kw OR 'sierra leone':ti,ab,kw OR somalia:ti,ab,kw OR 'south africa':ti,ab,kw OR 'south sudan':ti,ab,kw OR tanzania:ti,ab,kw OR togo:ti,ab,kw OR uganda:ti,ab,kw OR zambia:ti,ab,kw OR zimbabwe:ti,ab,kw OR 'ivory coast':ti,ab,kw

**Search results:** 16 September 2026 = **4 records**

**S1.8 APA PsycINFO**

**Database:** Embase
**Platform:** EBSCO Host
**Search date:** 16 September 2026
**Records retrieved:** 2
**Limits/filters:** No additional publication-date, language or study-design filter applied at the database-search stage.
**Search field:** Title or Abstract (XB)

**Controlled vocabulary**

APA PsycINFO does not provide a MeSH-style controlled vocabulary equivalent to PubMed. The search therefore relied on database-indexed text fields using free-text terms.

**Final PsycINFO search strategy**

XB (prison* OR prisoner* OR inmate* OR jail* OR gaol* OR incarcerat* OR detainee* OR detain* OR detention OR custod* OR "correctional facilit*" OR "correctional institution*” OR "correctional service*" OR "penal institution*" OR penitentiary OR penitentiaries OR remand OR convict* OR offender* OR "prison population*" OR "incarcerated person*" OR "incarcerated people")

AND

XB ("eye disease*" OR "ocular disease*" OR "ophthalmic disease*" OR "ocular morbid*" OR "eye disorder*" OR "ocular disorder*" OR "ophthalmic disorder*" OR "visual impair*" OR "vision impair*" OR "low vision" OR blindness OR blind OR "eye condition*" OR "ocular condition*" OR "refractive error*" OR ametropi* OR myopi* OR hyperopi* OR hypermetropi* OR astigmati* OR presbyopi* OR cataract* OR glaucoma* OR conjunctivit* OR trachoma* OR pterygi* OR keratit* OR "corneal opacit*" OR uveitis OR retinopath* OR "diabetic retinopathy" OR "retinal disease*" OR "optic neuropath*" OR maculopath* OR "eye infection*" OR ophthalmolog* OR "eye health" OR "ocular health")

AND

XB (“Africa South of the Sahara" OR "sub-Saharan Africa" OR SSA OR "West Africa" OR "East Africa" OR "Central Africa " OR "southern Africa" OR Angola OR Benin OR Botswana OR "Burkina Faso" OR Burundi OR "Cabo Verde" OR "Cape Verde" OR Cameroon OR "Central African Republic" OR Chad OR Comoros OR "Republic of the Congo" OR "Congo Brazzaville" OR "Democratic Republic of the Congo" OR DRC OR "Congo Kinshasa" OR Djibouti OR "Equatorial Guinea" OR Eritrea OR Eswatini OR Ethiopia OR Gabon OR Gambia OR Ghana OR Guinea OR "Guinea-Bissau" OR Liberia OR Kenya OR Lesotho OR Madagascar OR Malawi OR Mali OR Mauritania OR Mauritius OR Mozambique OR Namibia OR Niger OR Nigeria OR Rwanda OR "Sao Tome and Principe" OR Senegal OR Seychelles OR "Sierra Leone" OR Somalia OR "South Africa" OR "South Sudan" OR Tanzania OR Togo OR Uganda OR Zambia OR Zimbabwe OR "Cote d'Ivoire" OR "Côte d'Ivoire" OR "Ivory Coast")

**Search results:** 16 September 2026 = **2 records**

**S1.9 CINAHL**

**Database:** CINAHL
**Platform:** EBSCO Host
**Search date:** 16 September 2026
**Records retrieved:** 1
**Limits/filters:** No additional publication-date, language or study-design filter applied at the database-search stage.
**Search field:** Title or Abstract (XB)

**Controlled vocabulary**

CINAHL does not provide a MeSH-style controlled vocabulary equivalent to PubMed. The search therefore relied on database-indexed text fields using free-text terms.

**Final CINAHL search strategy**

XB (prison* OR prisoner* OR inmate* OR jail* OR gaol* OR incarcerat* OR detainee* OR detain* OR detention OR custod* OR "correctional facilit*" OR "correctional institution*” OR "correctional service*" OR "penal institution*" OR penitentiary OR penitentiaries OR remand OR convict* OR offender* OR "prison population*" OR "incarcerated person*" OR "incarcerated people")

AND

XB ("eye disease*" OR "ocular disease*" OR "ophthalmic disease*" OR "ocular morbid*" OR "eye disorder*" OR "ocular disorder*" OR "ophthalmic disorder*" OR "visual impair*" OR "vision impair*" OR "low vision" OR blindness OR blind OR "eye condition*" OR "ocular condition*" OR "refractive error*" OR ametropi* OR myopi* OR hyperopi* OR hypermetropi* OR astigmati* OR presbyopi* OR cataract* OR glaucoma* OR conjunctivit* OR trachoma* OR pterygi* OR keratit* OR "corneal opacit*" OR uveitis OR retinopath* OR "diabetic retinopathy" OR "retinal disease*" OR "optic neuropath*" OR maculopath* OR "eye infection*" OR ophthalmolog* OR "eye health" OR "ocular health")

AND

XB (“Africa South of the Sahara" OR "sub-Saharan Africa" OR SSA OR "West Africa" OR "East Africa" OR "Central Africa " OR "southern Africa" OR Angola OR Benin OR Botswana OR "Burkina Faso" OR Burundi OR "Cabo Verde" OR "Cape Verde" OR Cameroon OR "Central African Republic" OR Chad OR Comoros OR "Republic of the Congo" OR "Congo Brazzaville" OR "Democratic Republic of the Congo" OR DRC OR "Congo Kinshasa" OR Djibouti OR "Equatorial Guinea" OR Eritrea OR Eswatini OR Ethiopia OR Gabon OR Gambia OR Ghana OR Guinea OR "Guinea-Bissau" OR Liberia OR Kenya OR Lesotho OR Madagascar OR Malawi OR Mali OR Mauritania OR Mauritius OR Mozambique OR Namibia OR Niger OR Nigeria OR Rwanda OR "Sao Tome and Principe" OR Senegal OR Seychelles OR "Sierra Leone" OR Somalia OR "South Africa" OR "South Sudan" OR Tanzania OR Togo OR Uganda OR Zambia OR Zimbabwe OR "Cote d'Ivoire" OR "Côte d'Ivoire" OR "Ivory Coast")

**Search results:** 16 September 2026 = **1 record**

**S1.7 Search update procedure**

The original search was conducted on 21 March 2026. In response to peer-review, an updated search was conducted on 16 September 2026.

The updated search retrieved:

- PubMed: **16 records**
- Scopus: **24 records**
- Web of Science Core Collection: **61 records**
- Google Scholar: **861 records**
- **AJOL = 8 records**
- **Embase = 4 records**
- **APA PsycINFO = 2 records**
- **CINAHL = 1 record**

**S1.8 Record management and deduplication**

Records retrieved from all information sources were exported in the available bibliographic format and combined in **EndNote 21**.

Duplicate records were identified using automated duplicate detection followed by manual verification of potentially ambiguous matches. Duplicates identified within individual search sources and duplicates arising between databases were removed. The deduplicated record set was subsequently transferred to **Covidence** for study screening and selection.

Where the same publication was retrieved from more than one database or search source, it was retained as a single bibliographic record for screening. Where multiple reports referred to the same underlying study, the reports were linked and treated as reports of the same study during study selection and synthesis.

The number of duplicate records removed was recorded and used to reconcile the search audit table with the PRISMA flow diagram.

**S1.10 Language restriction**

The eligibility criteria restricted included publications to **English-language studies**. This restriction was applied during study selection rather than as a database-search filter.

The authors recognize that restricting eligibility to English-language publications may have introduced **language bias**. This limitation is potentially important for a review of Sub-Saharan Africa because relevant studies may have been published in French, Portuguese, or other languages, particularly from Francophone and Lusophone countries. Consequently, the possibility that relevant non-English evidence was not captured or included cannot be excluded. This limitation has been explicitly acknowledged.
