# Supplementary material for: Burden, spectrum and associated factors of ocular disease among incarcerated populations in sub-Saharan Africa: a systematic review and meta-analysis
Source: Front Med (Lausanne). 2026 Oct 23;13:1969779. doi: 10.3389/fmed.2026.1969779 (PMC13741224; doi:10.3389/fmed.2026.1969779)
Supplement: Supplementary file 2 [file Supplementaryfile5.docx]

**S5.1 Risk of bias for included studies**

**S5.2 Quality assessment using NIH quality assessment for observational and cross-sectional studies**

| **Author, Year** | **Q1. Was the research question or objective in this paper clearly stated?** | **Q2. Was the study population clearly specified and defined?** | **Q3. Was the participation rate of eligible persons at least 50%?** | **Q4. Were all the subjects selected or recruited from the same or similar populations (including the same time period)? Were inclusion and exclusion criteria for being in the study prespecified and applied uniformly to all participants?** | **Q5. Was a sample size justification, power description, or variance and effect estimates provided?** | **Q6. For the analyses in this paper, were the exposure(s) of interest measured prior to the outcome(s) being measured?** | **Q7. Was the timeframe sufficient so that one could reasonably expect to see an association between exposure and outcome if it existed?** | **Q8. For exposures that can vary in amount or level, did the study examine different levels of the exposure as related to the outcome (e.g., categories of exposure, or exposure measured as continuous variable)?** | **Q9. Were the exposure measures (independent variables) clearly defined, valid, reliable, and implemented consistently across all study participants?** | **Q10. Was the exposure(s) assessed more than once over time?** | **Q11. Were the outcome measures (dependent variables) clearly defined, valid, reliable, and implemented consistently across all study participants?** | **Q12. Were the outcome assessors blinded to the exposure status of participants?** | **Q13. Was loss to follow-up after baseline 20% or less?** | **Q14. Were key potential confounding variables measured and adjusted statistically for their impact on the relationship between exposure(s) and outcome(s)?** | **Overall Quality Rating** |
| --- | --- | --- | --- | --- | --- | --- | --- | --- | --- | --- | --- | --- | --- | --- | --- |
| Mwakakonyole et al. 2024 | Yes | Yes | Yes | Yes | Yes | No | NA | Yes | Yes | No | Yes | NR | NA | Yes | Good |
| Zalwango et al. 2021 | Yes | Yes | Yes | Yes | Yes | No | NA | Yes | Yes | No | Yes | NR | NA | Yes | Good |
| Ajite et al. 2011 | Yes | Yes | Yes | Yes | NR | No | NA | NR | Yes | No | Yes | NR | NA | No | Fair |
| Ohikhuare 2020 | Yes | Yes | CD | No | NR | No | NA | No | CD | No | CD | NR | NA | No | Poor |
| Ekwenchi et al. 2009 | Yes | Yes | Yes | Yes | NR | No | NA | NA | Yes | No | Yes | NR | NA | Yes | Good |
| Mathenge et al 2007 | Yes | Yes | Yes | Yes | NR | No | NA | Yes | Yes | No | Yes | NR | NA | No | Fair |

**S5.3. GRADE assessment for main outcomes**

| **Certainty assessment** | | | | | | | **Impact** | **Certainty** | **Importance** |
| --- | --- | --- | --- | --- | --- | --- | --- | --- | --- |
| **№ of studies** | **Study design** | **Risk of bias** | **Inconsistency** | **Indirectness** | **Imprecision** | **Other considerations** |  |  |  |
| **Overall pooled prevalence of ocular diseases** | | | | | | | | | |
| 6 | non-randomised studies | serious^a^ | serious^b^ | serious^c^ | serious^d^ | none | No of patients: 3,361  Pooled Prevalence = 43.1% (95% CI: 12.4% to 77.1%) | ⨁◯◯◯ Very low^a,b,c,d^ | CRITICAL |
| **Prevalence of Conjunctivitis** | | | | | | | | | |
| 4 | non-randomised studies | serious^e^ | serious^b^ | serious^c^ | serious^f^ | none | No of patients: 1,124  Pooled Prevalence = 23.9% (95% CI: 10.7% to 40.1%) | ⨁◯◯◯ Very low^b,c,e,f^ | CRITICAL |
| **Prevalence of Refractive Error** | | | | | | | | | |
| 4 | non-randomised studies | serious^e^ | serious^b^ | serious^c^ | serious^g^ | none | No of patients: 1,124  Pooled Prevalence = 19.5% (95% CI: 12.5% to 27.5%) | ⨁◯◯◯ Very low^b,c,e,g^ | CRITICAL |
| **Prevalence of Presbyopia** | | | | | | | | | |
| 3 | non-randomised studies | not serious | serious^b^ | serious^c^ | very serious^h^ | none | No of patients: 1,048  Pooled Prevalence = 18.6% (95% CI: 5.2% to 37.7%) | ⨁◯◯◯ Very low^b,c,h^ | CRITICAL |
| **Prevalence of Posterior segment eye diseases** | | | | | | | | | |
| 4 | non-randomised studies | serious^e^ | very serious^b^ | serious^c^ | very serious^i^ | none | No of patients: 793  Pooled Prevalence = 9.8% (95% CI: 2.5% to 20.7%) | ⨁◯◯◯ Very low^b,c,e,i^ | CRITICAL |
| **Prevalence of Pterygium** | | | | | | | | | |
| 3 | non-randomised studies | not serious | serious^b^ | serious^c^ | very serious^j^ | none | No of patients: 1,048  Pooled Prevalence = 9.3% (95% CI: 2.5% to 19.4%) | ⨁◯◯◯ Very low^b,c,j^ | CRITICAL |
| **Prevalence of Cataract** | | | | | | | | | |
| 4 | non-randomised studies | not serious | serious^b^ | serious^c^ | serious^k^ | none | No of patients: 1,070  Pooled Prevalence = 7.4% (95% CI: 3.8% to 11.8%) | ⨁◯◯◯ Very low^b,c,k^ | CRITICAL |
| **Prevalence of Glaucoma** | | | | | | | | | |
| 3 | non-randomised studies | not serious | serious^b^ | serious^c^ | very serious^l^ | none | No of patients: 717  Pooled Prevalence = 5.6% (95% CI: 0.6% to 14.2%) | ⨁◯◯◯ Very low^b,c,l^ | CRITICAL |
| **Prevalence of Corneal scar/opacity** | | | | | | | | | |
| 5 | non-randomised studies | not serious | serious^m^ | serious^c^ | serious^n^ | none | No of patients: 1,317  Pooled Prevalence = 3.0% (95% CI: 1.3% to 5.2%) | ⨁◯◯◯ Very low^c,m,n^ | CRITICAL |

**CI:** confidence interval

#### **Explanations**

a. 5/6 studies high quality, but the one low-quality study meaningfully shifts the pooled estimate on leave-one-out (43.1%→33.8%)

b. Extremely wide I²>75%

c. Standard cross-study concern

d. Only k=6 studies; 95% CI 12.4–77.1%; 95% PI 0.3–99.7%

e. Includes a low methodological quality study

f. CI (10.7–40.1%) spans nearly 4-fold, and the outlier study visibly widens this interval

g. CI (12.5–27.5%) spans roughly a 2.2-fold range around the point estimate

h. CI (5.2–37.7%) spans more than a 7-fold range

i. CI (2.5–20.7%) spans over 8-fold, driven by the outlier study's wide individual CI (32.9–74.7%)

j. CI (2.5–19.4%) spans nearly 8-fold

k. CI (3.8–11.8%) spans about 3.1-fold

l. CI (0.6–14.2%) is extremely wide relative to the point estimate (nearly 24-fold), lower bound approaches zero

m. Moderate heterogeneity I²=67.3%

n. CI (1.3–5.2%) spans 4-fold
